# Herpes Simplex Virus 1 Us3 Deletion Mutant is Infective Despite Impaired Capsid Translocation to the Cytoplasm

**DOI:** 10.3390/v7010052

**Published:** 2015-01-12

**Authors:** Peter Wild, Sabine Leisinger, Anna Paula de Oliveira, Elisabeth M. Schraner, Andres Kaech, Mathias Ackermann, Kurt Tobler

**Affiliations:** 1 Institute of Veterinar Anatomy, Winterthurerstrasse 260, CH-8057 Zürich, Switzerland; E-Mails: sabineleisinger@yahoo.de (S.L.); emschra@vetanat.uzh.ch (E.M.S.); 2 Institute of Virology, Winterthurerstrasse 266a, CH-8057 Zürich, Switzerland; E-Mails: annapaula.oliveira@gmail.com (A.P.-O.); Mathias.Ackermann@vetvir.uzh.ch (M.A.); Kurt.Tobler@vetvir.uzh.ch (K.T.); 3 Center for Microscopy and Image Analysis, Winterthurerstrasse 190,CH-8057 Zürich, Switzerland; E-Mail: andres.kaech@zmb.uzh.ch

**Keywords:** HSV-1, Us3 protein kinase, envelopment, morphogenesis, electron microscopy, morphometry

## Abstract

Herpes simplex virus 1 (HSV-1) capsids are assembled in the nucleus bud at the inner nuclear membrane into the perinuclear space, acquiring envelope and tegument. In theory, these virions are de-enveloped by fusion of the envelope with the outer nuclear membrane and re-enveloped by Golgi membranes to become infective. Us3 enables the nucleus to cytoplasm capsid translocation. Nevertheless, Us3 is not essential for the production of infective progeny viruses. Determination of phenotype distribution by quantitative electron microscopy, and calculation per mean nuclear or cell volume revealed the following: (i) The number of R7041(∆U_S_3) capsids budding at the inner nuclear membrane was significantly higher than that of wild type HSV-1; (ii) The mean number of R7041(∆U_S_3) virions per mean cell volume was 2726, that of HSV-1 virions 1460 by 24 h post inoculation; (iii) 98% of R7041(∆U_S_3) virions were in the perinuclear space; (iv) The number of R7041(∆U_S_3) capsids in the cytoplasm, including those budding at Golgi membranes, was significantly reduced. Cell associated R7041(∆U_S_3) yields were 2.37 × 10^8^ and HSV-1 yields 1.57 × 10^8^ PFU/mL by 24 h post inoculation. We thus conclude that R7041(∆U_S_3) virions, which acquire envelope and tegument by budding at the inner nuclear membrane into the perinuclear space, are infective.

## 1. Introduction

Herpes simplex virus 1 (HSV-1) is composed of four morphologically distinct substructures: the core containing the viral genomic DNA, the icosahedral capsid, the tegument surrounding the capsid, and the viral envelope with embedded glycoproteins [1]. Capsids are formed in replication centers (RCs) [2] in host cell nuclei, and transported to the nuclear periphery. Morphogenesis of virions and their transportation to the cell periphery for release into the extracellular space is still under debate [1]. Capsids bud at the inner nuclear membrane (INM) acquiring tegument and envelope. The result is a fully enveloped virion located in the perinuclear space (PNS) [3]. The envelope is assumed to fuse with the outer nuclear membrane (ONM) releasing capsid and tegument into the cytoplasmic matrix [4,5]. According to the dogma, these capsids need to be re-enveloped by budding at membranes of the trans-Golgi network to become infectious virions [3,4,6]. Interestingly, virions were repeatedly demonstrated within the rough endoplasmic reticulum (RER) indicating intraluminal virus transportation out of the PNS into adjacent RER cisternae [7,8,9,10,11,12,13]. This fact questions the correctness of the de-re-envelopment theory simply because membranes of particles cannot have the ability for fusion with membranes they are transported along: The viral membrane can either fuse with any membranes it becomes in close apposition or the fusion ability is prevented so that the virion can be transported.

Virions may accumulate in the PNS late in infection [14] or in the absence of gB [15]. HSV-1 deleted of the Us3 gene (HSV-1∆U_S_3), which encodes a protein kinase (Us3), also accumulate in the PNS and in invaginations formed by nuclear membranes [16,17] suggesting that Us3 is involved in fusion of the viral envelope with the ONM. Phosphorylation of gB [18], a member of the quartet of glycoproteins responsible for fusion during cell entry [19] is considered to play a key role in the fusion process at the ONM. Despite the fusion inability at the ONM [18] Us3 deletion mutants produce infective progeny virus in cell cultures [16,20] raising the question when and where Us3 deletion mutants become infective. To get an idea about the fate of virions derived by budding at the INM in the absence of Us3, we investigated the morphogenesis of the Us3 deletion mutant R7041(∆U_S_3) [21,22] in Vero cells by quantitative transmission electron microscopy employing a technique for rapid freezing followed by freeze-substitution that lead to improved retention of lipids [23], and improved preservation of cellular architecture [24] enabling visualization of biological structures in a state that is closest to the situation in living cells [25]. We will show that a single cell produces ~2700 enveloped R7041∆U_S_3 virions in average. 98% of them are retained in the PNS by 24 h post inoculation (hpi).

## 2. Materials and Methods

### 2.1. Cells and Viruses

Vero cells (European Collection of Cell Cultures) were grown in Dulbecco’s modified minimal essential medium (DMEM; Gibco, Bethesda, MD, USA) supplemented with penicillin (100 U/mL), streptomycin (100 μg/mL) and 10% fetal bovine serum (FBS; Gibco). The Us3 deletion mutant R7041(∆U_S_3) and the repair mutant R2641 were kindly provided by B. Roizman, University of Chicago, IL, USA. Wild-type (wt) HSV-1 strain F, R7041(∆Us3) and R2641 were propagated in Vero cells. Virus yields were determined by plaque titration.

### 2.2. Virus Replication Assays

Confluent Vero cells grown in 12-well plates were inoculated at a multiplicity of infection (MOI) of 5 plaque forming units (PFU) per cell. After 2 h of incubation at 37 °C, 5% CO_2_, the cultures were washed 3 times with PBS and then incubated with DMEM containing 2% FBS. Samples (cells and cell culture medium) were removed after 0, 9, 12, 16, 20 and 24 h. Cell culture medium was removed from the cells and serially diluted to determine the titers on Vero cells. The titers of cell-associated virus were determined on Vero cells inoculated with serial dilutions of supernatants of cells prepared by 3 cycles of freezing and thawing, followed by centrifugation at 1’900 g.

### 2.3. Cryo-Fixation for Transmission Electron Microscopy

Fifty-micrometer-thick sapphire disks (Bruegger, Minusio, Switzerland) measuring 3 mm in diameter were coated with 8–10 nm carbon obtained by evaporation under high vacuum conditions to enhance cell growth. Vero cells were grown for 2 days on sapphire disks placed in 6 well plates. Cells were inoculated with R7041(∆Us3), R2641 or wt HSV-1 at a MOI of 5, incubated at 37 °C, and fixed at 9, 12, 16, 20 and 24 h post inoculation (hpi) by adding 0.25% glutaraldehyde to the medium prior to freezing in a high-pressure freezing unit (HPM010; BAL-TEC, Balzers, Liechtenstein) applying the sandwich method [26], and processed as described in detail [13,27]. In brief, the frozen water was substituted with acetone in a freeze-substitution unit (FS 7500; Boeckeler Instruments, Tucson, Arizona) at −88 °C with acetone and subsequently fixed with 0.25% glutaraldehyde and 0.5% osmium tetroxide raising the temperature gradually to +2 °C to achieve good contrast of membranes [28], and embedded in epon at 4 °C followed by polymerization at 60 °C for 2.5 days. Serial sections of 60 to 90 nm thickness were analyzed in a transmission electron microscope (CM12; Philips, Eindhoven, The Netherlands) equipped with a CCD camera (Ultrascan 1000; Gatan, Pleasanton, CA, USA) at an acceleration voltage of 100 kV.

### 2.4. Determination of Nuclear and Cell Volume

Cells were grown for 2 days on 0.17 mm thick cover slips measuring 12 mm in diameter (Assistent, Sondheim, Germany), inoculated with R7041(∆Us3), wt HSV-1 or the repair mutant R2641 at a MOI 5, and incubated at 37 °C for 9 to 24 h. After fixation with 2% formaldehyde for 25 min at room temperature, cells were washed with PBS and stained with 4',6-diamidino-2-phenylindol (DAPI). Stacks of images were taken by confocal laser scanning microscopy (SP2, Leica, Wetzlar, Germany), and deconvolved employing the deconvolution algorithm of the program suite Huygens Essential (SVI, Hilversum, The Netherlands).

Nuclei of Vero cells are triaxial ellipsoids. Therefore, the mean nuclear volume (V_n_) was calculated on the basis of the half axes (a, b, c) measured on 25 deconvolved confocal images of DAPI stained nuclei according to the equation V_n_ = $V=\frac{4}{3}\pi abc$ as described in detail recently [29]. The relation between nuclear and cell volume can be evaluated by electron microscopic morphometric analysis of thin sections. Then, the absolute mean cell volume can be calculated on the basis of the absolute mean nuclear volume. For this, 20 images were sampled at random per experiment on cryo-fixed cells of 5 independent experiments at a final magnification of 6300x. From these images, nuclear density (Vv_n_) and cytoplasmic density (Vv_cy_) were estimated applying the point counting method [30] according to the equations Vv_n_ = P_n_/(P_n_ + P_cy_) and Vv_cy_ = P_cy_/(P_n_ + P_cy_), whereby P_n_ are points hitting the nucleus, and P_cy_ are points hitting the cytoplasm. On the basis of the absolute nuclear volume obtained from confocal images, the absolute mean cell volume (V_c_) was calculated as V_c_ = V_n_/Vv_n_.

### 2.5. Phenotype Distribution

Virus particles—including capsids budding at the INM and ONM, virions in the PNS, virions in the RER, capsids in the cytoplasm, involved in wrapping or in budding at Golgi membranes, virions in Golgi cisternae and/or vacuoles—were counted on TEM images selected at random at each time point (9, 12, 16, 20, 24 hpi). Then the nuclear area was estimated by point counting applying a multipurpose test system [30]. The mean nuclear area (A_n_) was calculated using the equation A_n_ = P_n_∙d^2^, whereby P_n_ are points hitting the nuclei and d the test line length. From the number of particles (p) and the nuclear area, the numerical density N_Vp_ = p/(A_n_)/D can be calculated, whereby D is the mean particle diameter: D = 125 nm for capsids [31]; 200 nm for virions [32]. Then, the absolute number of particles (N_p_) per mean nuclear volume can be calculated: N_p_ = N_Vp_∙V_n_. This was applied for budding capsids at the INM and ONM, and for virions in the PNS. Similarly, the numerical density of virus particles in the cytoplasm can be calculated: N_Vp_ = p/(A_cy_)/D. The absolute number of virus particles is N_p_ = N_Vp_∙V_c_.

### 2.6. Cryo-Field Emission Scanning Electron Microscopy (cryo-FESEM) 

Cells were grown in 25 cm^2^ cell culture flasks for 2 days prior to inoculation. Cells were harvested 16 hpi by trypsinization followed by centrifugation at 150 x g for 8 min. The pellet was re-suspended in 1 mL fresh medium, collected in Eppendorf tubes and fixed by adding 0.25% glutaraldehyde to the medium. The suspension was kept in the tubes at 4 °C until cells were sedimented. Then, cells were frozen in a high-pressure freezing machine (EM HPM100, Leica Microsystems, Vienna, Austria) and prepared as described in detail [33,34]. In brief, frozen cells were fractured at −120 °C in a freeze-fracturing device BAF 060 (Leica Microsystems) in a vacuum of 10^−7^ mbar. The fractured surfaces were partially freeze-dried (“etched”) at −105 °C for 2 min, and coated with 2 nm platinum/carbon by electron beam evaporation at angle of 45°. Then, specimens were coated additionally with 4 nm carbon to reduce electron beam damaging during imaging at high magnifications. Specimens were imaged in an Auriga 40 Cross Beam system (Zeiss, Oberkochen, Germany) equipped with a cryo-stage at −115 °C and an acceleration voltage of 5 kV using the inlens secondary electron detector.

## 3. Results

### 3.1. Budding of R7041(∆Us3) Capsids at the Inner Nuclear Membrane

**Figure 1:**
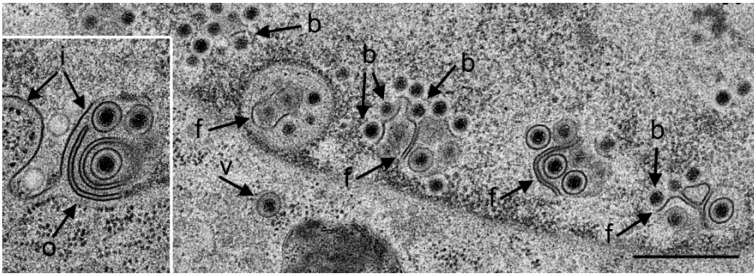
TEM of a Vero cell 12 hpi with R7041(∆Us3). Many capsids are at the nuclear periphery, some of them bud (b) at the inner nuclear membrane (INM) (i). The INM formed folds that penetrate into the nucleus clearly seen in the inset. Fully enveloped virions are located adjacent to folds (f), and one in the rough endoplasmic reticulum (RER) (v). The outer nuclear membrane (ONM) (o) is only clearly visible in the inset. Bar = 500 nm.

**Figure 2:**
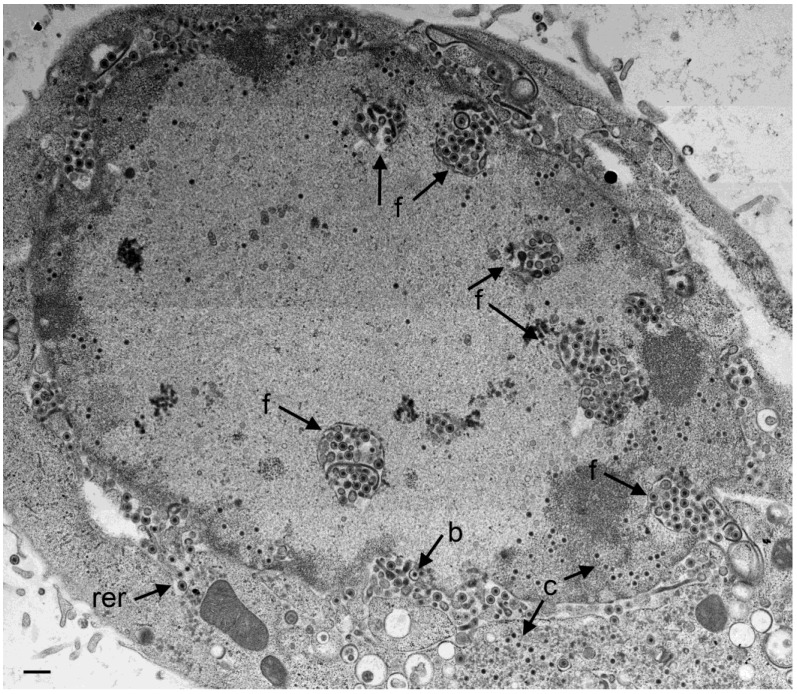
TEM of a Vero cell 24 hpi with R7041(∆Us3). Large number of virions are located in the PNS and in invaginations (f) formed by folding of the INM into the nucleus, and in “vacuoles” often referred to as herniations. They are considered likely to represent invaginations of the INM as easily apparent at the right bottom. A few virions are in the RER (rer) adjacent to the perinuclear space (PNS). Numerous capsids (c) are distributed in the nucleus and some in the cytoplasm. A few capsids bud (b) at the INM. Bar = 500 nm.

The Us3 enables translocation of capsids from the nucleus to the cytoplasm. Capsids derived from budding at the INM accumulate in invaginations formed by nuclear membranes in cells infected with HSV-1 [16,17] or pseudorabies virus Us3 deletion mutants [35,36]. TEM revealed that the INM formed folds starting prior to 9 hpi with R7041(∆Us3). Numerous R7041(∆Us3) capsids bud at membrane folds (Figure 1). The INM formed invaginations when infection proceeded. Such invaginations were filled with virions by 24 hpi (Figure 2).

**Figure 3:**
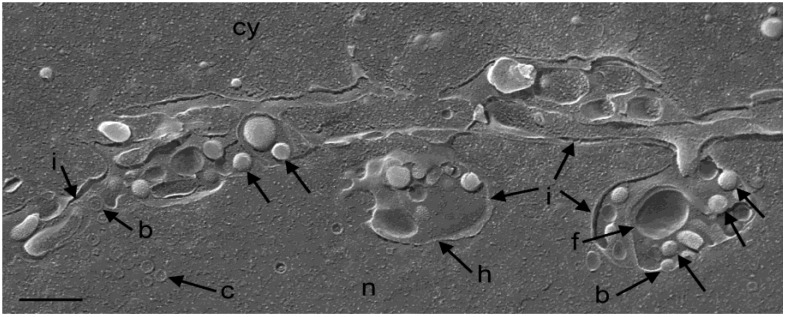
Cryo-FESEM of a R7041(∆Us3) infected Vero cell (12 hpi) showing a fracture plane through the nucleus (n) and the cytoplasm (cy). The INM (i) formed invaginations from which folds (f) arise. Membranes of herniations (h) continue into the INM. Capsids (c) bud (b) at the INM. Virions (arrows) are in invaginations and in the PNS. Bar = 500 nm.

To ascertain whether membranes forming “vacuoles” within nuclei in a given TEM image correspond to the INM, we employed cryo-FESEM. Imaging of cryo-fractured cells demonstrated that the INM invaginated into the nucleus and that the INM formed folds at such invaginations (Figure 3). The “vacuoles” (often referred to as herniations) in the nucleus are thus considered likely to represent invaginations of the INM which is also apparent at some sites in Figure 2. Here for simplicity, invaginations and herniations are referred to as PNS.

**Figure 4:**
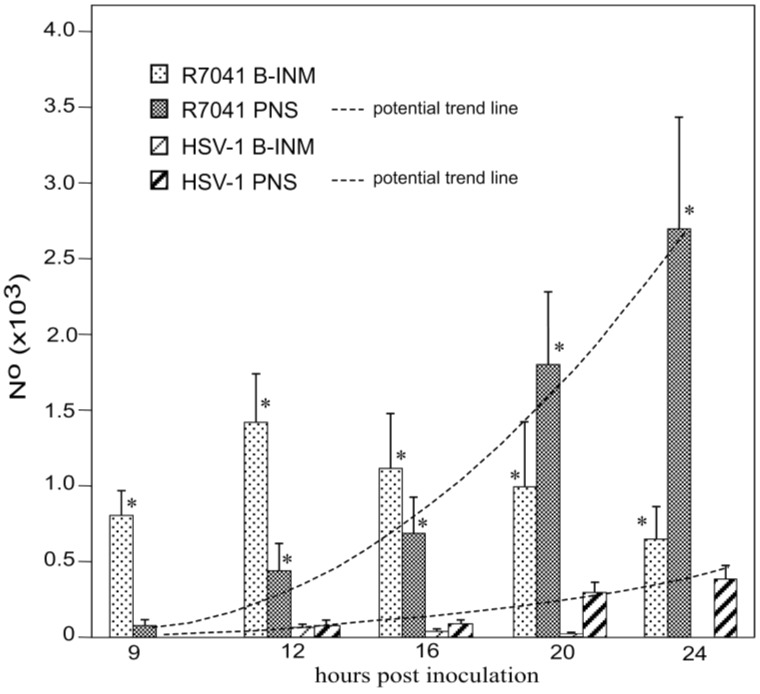
Mean and standard deviation of R7041(∆U_S_3) and wt HSV-1 capsids budding at the INM, and of virions within the PNS calculated per mean nuclear volume. Budding of R7041(∆U_S_3) capsids peaked at 12 hpi whereas the number of R7041(∆U_S_3) and wt HSV-1 virions potentially increased (potential trend lines) though with a difference of more than 2000 virions by 24 hpi. *p* < 0.0001, *n* = 5.

For quantitative analysis of virus particle distribution, we first calculated the mean nuclear volume. Nuclei in Vero cells grown as monolayers are ellipsoids as revealed by three dimensional re-constructions of confocal images. Therefore, the nuclear volume was calculated on the basis of the 3 nuclear axes measured in confocal microscopic images as reported previously [37]. Next, we determined the numerical density of capsids budding at the INM, and of virions within the PNS. Then we calculated the mean number of capsids budding at the INM and of virions within the PNS per mean nuclear volume (Figure 4). It turned out that the mean number of budding R7041(∆Us3) capsids at the INM increased from 800 (±160) at 9 hpi to 1390 (±230) at 12 hpi, and decreased to 632 (±198) at 24 hpi. The maximal number of budding wt HSV-1 capsids was 90 (±20) at 12 hpi, *i.e.*, by a factor of 15.4 lower than the number of budding R7041(∆Us3) capsids. Therefore, we conclude that R7041(∆Us3) capsids bud through the INM either at a slower pace than wt HSV-1 capsids or that budding activity of R7041(∆Us3) capsids is enhanced.

### 3.2. Retention of R7041(∆Us3) Virions in the PNS

Us3 is involved in the release of virions out of the PNS via fusion of the envelope with the ONM [16,18]. Therefore, we examined the PNS and the ONM. However, electron microscopy did not yield any indications for capsid release by fusion of the viral envelope with the ONM. Quantitative analysis revealed that the number of R7041(∆Us3) virions in the PNS steadily increased from 67 (±14) at 9 hpi to 2660 (±721) at 24 hpi (Figure 4). In contrast, the number of wt HSV-1 virions in the PNS was 97 (±31) at 12 hpi, and 390 (±82) at 24 hpi, respectively. The number of both R7041(∆Us3) and wt HSV-1 virions increased potentially. These data impressively confirm that Us3 plays a significant role in virion translocation out of the PNS.

### 3.3. Interaction of Capsids with the Outer Nuclear Membrane

**Figure 5:**
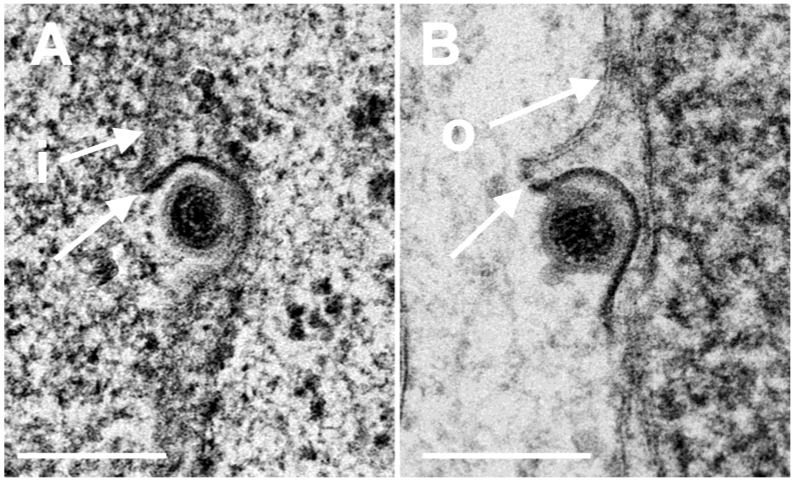
TEM images of budding capsids (**A**) at the INM (i) and (**B**) at the ONM (o). Note the identity of the two phenotypes, the ridge (arrows) formed on one side at each particle, and the eccentricity of the process. Ridges and eccentricity are not consistent with fusion. Bars 200 nm.

**Figure 6:**
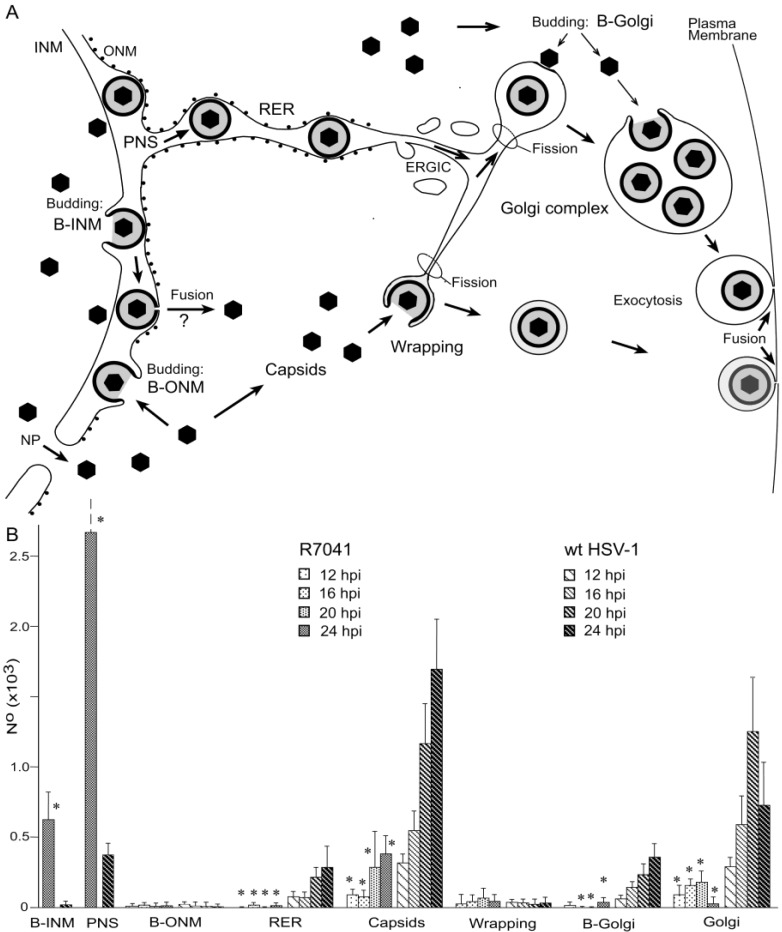
(**A**) Scheme of the envelopment pathways of HSV-1. Capsids bud at the INM (B-INM) and are postulated to fuse with the ONM. However, the process has all characteristic of budding (B-ONM). Capsids in the cytoplasm are transported to the Golgi complex where they bud into cisternae (B-Golgi) or they bud at Golgi membranes in such a manner that concomitantly transport vacuoles are formed (Wrapping). In both cases, the vacuolar membrane is dispatched from the donor membrane by fission. Alternatively, virions are transported from the PNS into RER cisternae. We postulate that they can be further transported into Golgi cisternae via the endoplasmic reticulum Golgi intermediate compartment (ERGIC) that connects the RER with the Golgi complex in herpes virus infected cells. Virions in transport vacuoles are released into the extracellular space by exocytosis. (**B**) Phenotype distribution of R7041(∆Us3) and HSV-1 envelopment. Mean total number and standard deviation of virus particles expressed per mean nuclear volume of budding capsids at the INM (B-INM) and of virions in the PNS (shown in more detail in Figure 4), and of capsids budding at the ONM (B-ONM). Virions in the RER, capsids in the cytoplasm (Capsids), capsids budding at Golgi membranes (B-Golgi), virions derived by wrapping (Wrapping), and virions in Golgi cisternae or vacuoles (Golgi) were expressed per mean cell volume. Data from R7041(∆Us3) infected cells are significantly different compared to wt HSV-1 infected cells (* *p* < 0.001, *n* = 5).

Interactions of capsids and tegument with the ONM have been described to our knowledge for the first time in baby hamster kidney cells infected by HSV-1 [38] four decades ago. The process was referred to as budding of capsids from the cytoplasm into the PNS. Indeed, all the processes encountered at the ONM were identical to those at the INM (Figure 5) and exhibited all characteristics for budding but none of fusion [39,40,41] (see also Figure 6). The number of capsid interactions with the ONM was low at any time. The highest number of R7041(∆Us3) capsids interacting with the ONM was 14 at 24 hpi, that of wt HSV-1 capsids was 19 at 12 hpi. Based on the identical phenotypes of the capsid interactions with the INM and ONM, we consider the capsid interaction at the ONM to be budding of capsids from the cytoplasm into the PNS rather than fusion of the viral envelope with the ONM.

### 3.4. Envelopment of R7041(∆U_S_3) Capsids at the Golgi Complex

**Figure 7:**
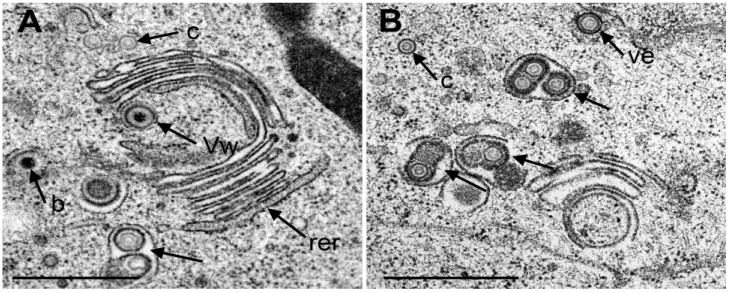
TEM images of Golgi fields in Vero cells at 12 hpi with wt HSV-1(A) and at 20 hpi (B) with the repair mutant R2641. (**A**) Close to a large Golgi stack is a virion in a concentric vacuole (Vw) derived by wrapping. Typically, the space between viral envelope and vacuolar membrane is similar in electron density as the tegument. In contrast, the space between viral envelope and membrane of vacuoles or Golgi cisternae containing one or more virions (arrow) derived either by budding into it or by intraluminal transportation from the RER. The space between virions and vacuolar membrane is electron lucent. (c) capsids, (b) budding capsid. Note that the outermost membrane of the Golgi stack shows the characteristic of RER membranes (rer) indicating connectivity between Golgi complex and RER. (**B**) Three vacuoles or Golgi cisternae (arrows) close to a small Golgi stack contain virions derived by budding or by intraluminal transportation. One virion is in the RER (Ve), and one capsid in the cytoplasm. Bar = 500 nm.

It was postulated that capsids need to be enveloped by Golgi membranes to become infective [4,6,42]. There are two ways of envelopment (Figure 6A). (i) Capsids bud at small Golgi cisternae pushing the membrane through the lumen towards the other membrane concomitantly forming the envelope and a transport vacuole (Figure 7A). This process has been designated as wrapping. Typically, the space between viral envelope and vacuolar membrane is similar in electron density as the tegument [13]; (ii) Capsids bud at membranes of large Golgi cisternae or vacuoles resulting in one or more virions in their electron lucent lumen (Figure 7B). It has to be born in mind that in a given section plane a vacuole might represent simply a part of a Golgi cisterna. The Golgi complex in Vero cells fragments and disperses in the course of HSV-1 infection [43]. In cells infected with wt HSV-1 or the repair mutant R2641, the Golgi complex consisted of typically arranged large stacks at 12 hpi (Figure 7A). Later in infection, small Golgi stacks were surrounded by vacuoles or dilated cisternae containing virions (Figure 7B).

**Figure 8:**
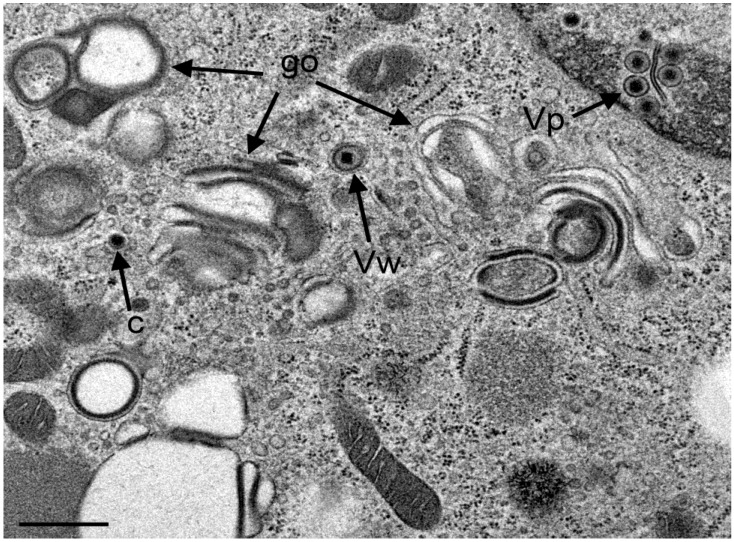
TEM images of R7041(∆U_S_3) infected Vero cells at 12 hpi showing three Golgi fields (go) with thick electron dense membranes, and a concentric vacuole enclosing a virion that resulted from wrapping (Vw). There are virions (Vp) in the PNS, and one capsid (c) in the cytoplasm. Bar = 500 nm.

If virus translocation out of the PNS is impaired in the absence of Us3 the question arises about the role of Golgi complex in envelopment of Us3 deletion mutants. Interestingly, the morphology of the Golgi complex in R7041(∆Us3) infected cells was entirely different from the Golgi complex in cells infected with wt HSV-1 or the repair mutant R2641. After R7041(∆Us3) infection, the Golgi complex comprised multiple stacks consisting often of thick electron dense membranes at the trans face by 12 hpi (Figure 8).

**Figure 9:**
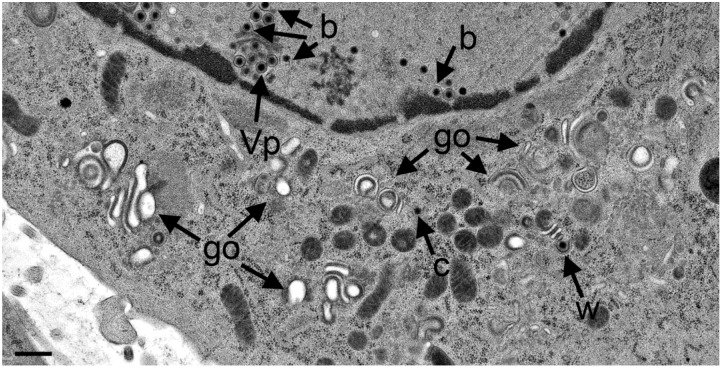
TEM images at 24 hpi with R7041(∆U_S_3). Multiple Golgi stacks (go) containing thick electron dense membranes, a single capsid (c) in the cytoplasm and one in the process of wrapping (w), virions in the PNS (Vp) and capsids budding (b) at the INM. Bar = 500 nm.

A few vacuoles derived by wrapping were in close vicinity. After 20 hpi, the Golgi complex comprised also multiple stacks consisting mostly of thick electron dense membranes (Figure 9).

**Figure 10:**
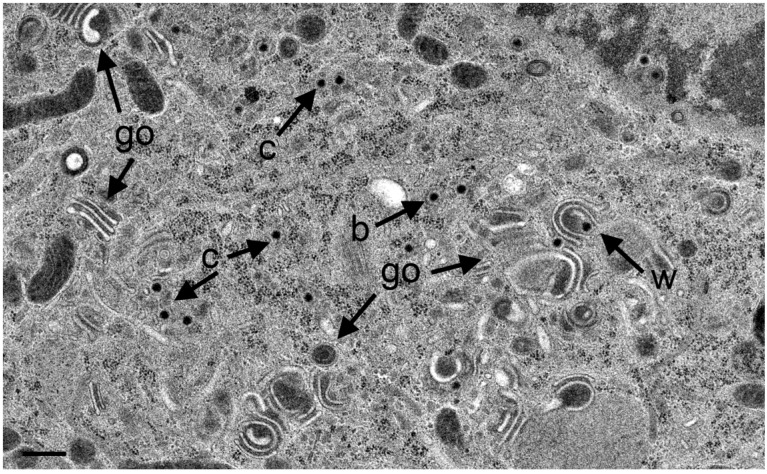
TEM images at 20 hpi with R7041(∆U_S_3) showing an area with at least 13 capsids (c), multiple Golgi stacks (go) containing thick electron dense membranes, and a capsid in the process of wrapping (w) that results in a malformed virion. One capsids bud (b) at a membrane that cannot be identified. Bar = 500 nm.

**Figure 11:**
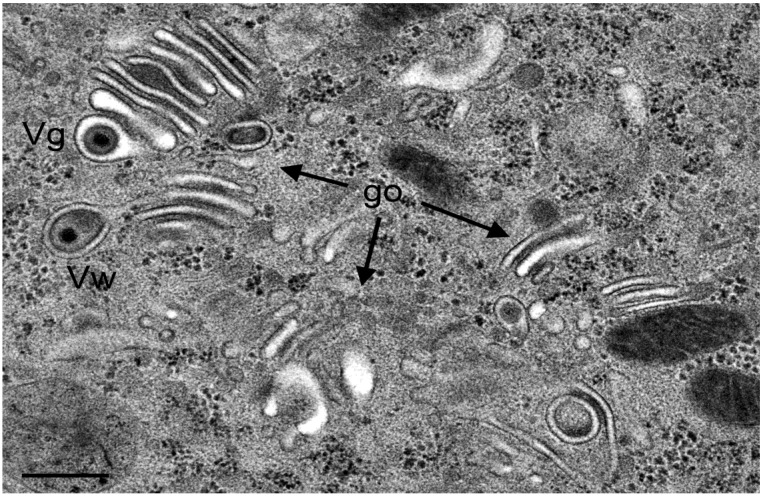
TEM images of three Golgi fields (go) containing thick electron dense membranes at 24 hpi with R7041(∆U_S_3). One virion has probably entered the cisterna by budding or via intraluminal transportation (Vg), the other virions has been probably derived by wrapping (Vw). Note the difference in electron density of the space between viral envelope and vacuolar membrane. Bar = 500 nm.

Sporadically, capsids were present in the cytoplasm between nucleus and Golgi fields (Figure 10). Some Golgi cisternae contained a virion (Figure 11).

This entirely different morphology of the Golgi complex upon R7041(∆Us3) infection prompted us to determine the number of capsids, of virions derived by wrapping and of virions in Golgi cisternae or and/or vacuoles. For this, we first evaluated the nuclear and cytoplasmic density for calculation of the mean cell volume as described recently [29,44]. Then, we determined the numerical density of virus particles in the cytoplasm without contact to membranes, and calculated their number per mean cell volume (Figure 6B). The data show that the mean number of R7041(∆Us3) capsids was up to 12 times (16 hpi) lower than that of wt HSV-1 capsids indicating that some R7041(∆Us3) capsids gained access to the cytoplasm despite impaired transportation out of the PNS.

The number of capsids in the process of wrapping and virions derived by wrapping was low. The maximum was 61 at 20 hpi, the minimum 27 at 12 hpi with R7041(∆Us3). After infection with wt HSV-1, the mean number of capsids involved in wrapping ranged from 32 to 40 at 16 and 20 hpi, respectively. In contrast, high numbers of wt HSV-1 capsids were involved in budding at Golgi membranes. Even more virions were within Golgi cisternae or vacuoles. The number of budding R7041(∆Us3) capsids was about 12 times lower, e.g., at 20 hpi, whereas the number of virions within Golgi cisternae and/or vacuoles was only 7 times lower than after wt HSV- 1 infection. Surprisingly, wt HSV-1 virions occurred up to 135 times more frequently within RER cisternae than R7041(∆Us3) virions. From these data, we conclude that (i) budding activity of capsids at Golgi membranes is drastically reduced in the absence of Us3; (ii) wrapping is not the main pathway in envelopment by Golgi membranes in wt HSV-1 or R7041(∆Us3) infected Vero cells; and (iii) intraluminal transportation is inhibited in the absence of Us3.

### 3.5. Infectious Progeny R7041(∆Us3) Virus

**Figure 12:**
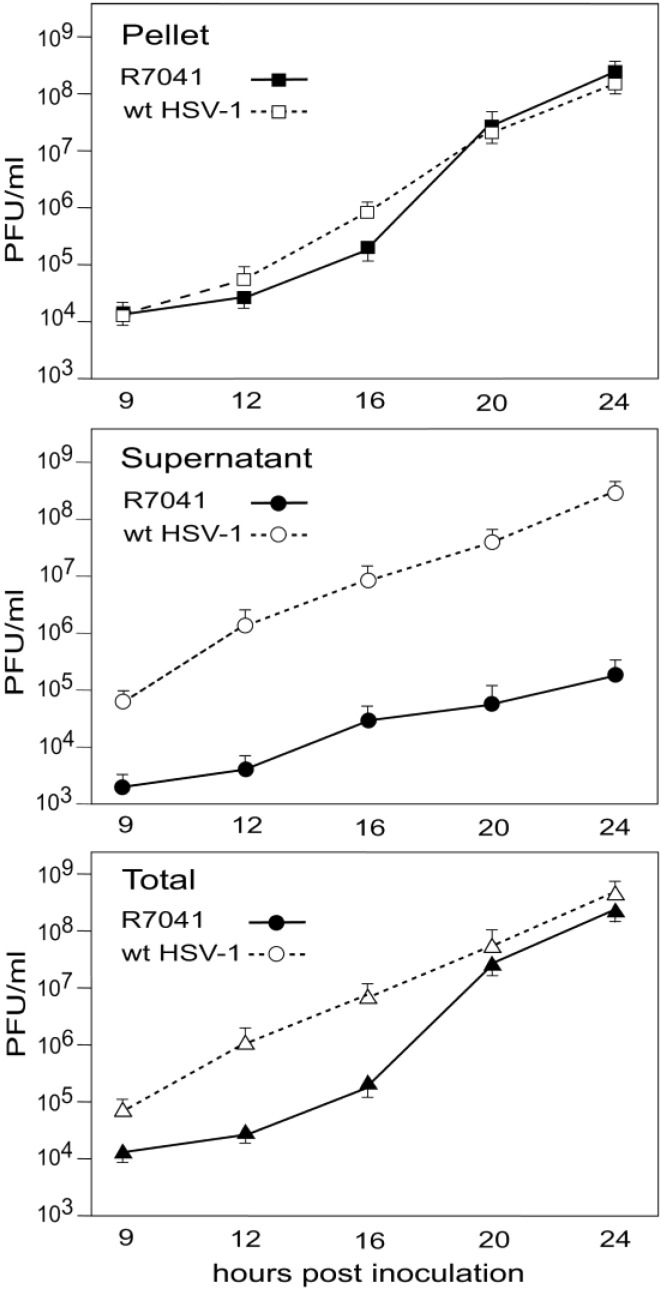
One-Step growth curves of R7041(∆Us3) and wt HSV-1. Cells were harvested at times post inoculation as indicated. Infectious virus yields in pellets and supernatants were determined by plaque titration. Mean and standard deviation were calculated from 4 experiments.

**Figure 13:**
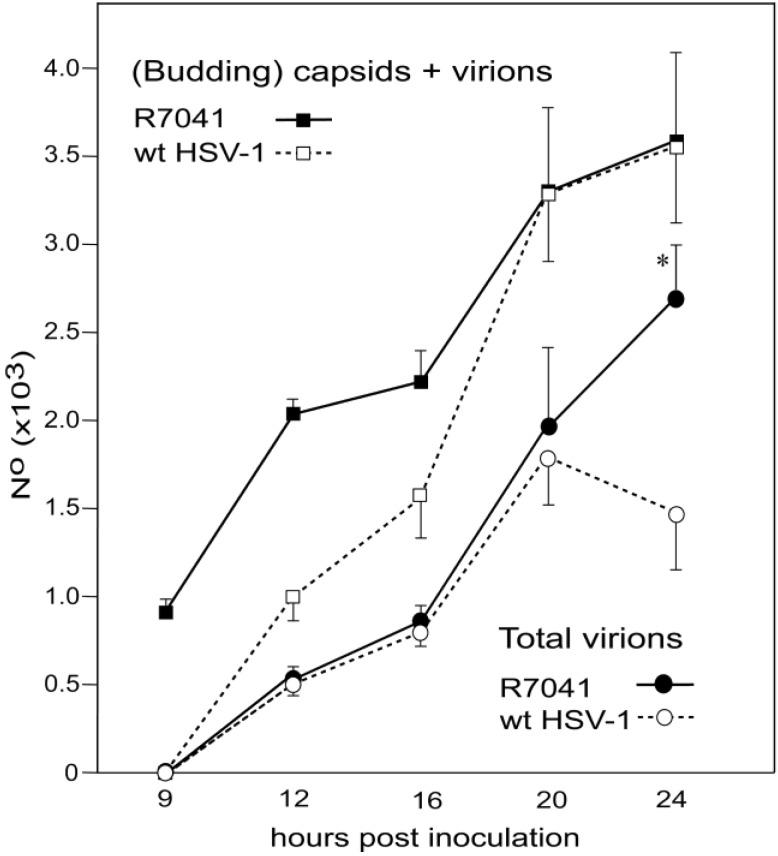
Mean total number and standard deviation of R7041(∆Us3) and wt HSV-1 virus particles (capsids in the cytoplasmic matrix, budding capsids at any membrane as well as virions), and of virions including those in the PNS, RER, Golgi cisternae and/or vacuoles. (* *p* < 0.001, *n* = 5).

HSV-1∆Us3 virions are infective though with delayed onset and reduced peaks of virus titers in HEp-2 cells [16] but with similar titers to wt HSV-1 in Vero cells [20]. Virus replication assays revealed also delay of R7041(∆Us3) growth (Figure 12). Infective virions were to a large extent cell associated. The mean total yield of R7041(∆Us3) at 24 hpi was 2.368 × 10^8^ PFU/mL, that of wt HSV-1 4.54 × 10^8^ PFU/mL. The intracellular yield of R7041(∆Us3) was 2.366 × 10^8^ PFU/mL, that of wt HSV-1 1.571 × 10^8^ PFU/mL, *i.e.*, 1.5 times higher. The total number of R7041(∆Us3) virus particles (capsids budding at any membrane as well as capsids in the cytoplasm and all virions) was equal to the total number of wt HSV-1 virus particles at 24 hpi (Figure 13). However, the total number of R7041(∆Us3) virions was almost twice as high as that of wt HSV-1 virions by 24 hpi. 97.7% of R7041(∆Us3) virions were localized in the PNS at 24 hpi. Therefore, we conclude that (i) R7041(∆Us3) virions in the PNS are infective, as proposed earlier [44]; and (ii) almost all infectious virions acquire envelope and tegument including all essential proteins by budding at the INM.

## 4. Discussion

### 4.1. Virions Derived by Budding at the INM

Envelopment of HSV-1 involves translocation of capsids from the nucleus to the cytoplasm, acquisition of tegument and the viral envelope including embedded glycoproteins, and finally transportation to the cell periphery for exocytotic release. Budding of capsids at the INM leads to fully enveloped virions located in the PNS [3,14]. It is assumed that these perinuclear virions are then de-enveloped by fusion of the envelope with the ONM releasing capsids and tegument into the cytoplasmic matrix. Infectious virions are finally generated by re-envelopment of capsids by budding at Golgi membranes [4,6]. Virions are assumed only to acquire essential proteins by budding at membranes of the *trans*-Golgi network [45] as deduced from immunogold labeling with antibodies against diverse proteins. However, to prove differences in labeling intensity, highly different sampling sizes between intracellular and extracellular HSV-1 virions were used for statistical analysis [42]. Some immunogold labeling studies in pseudorabies virus infected rabbit kidney cells were performed with poor resolution and poor preservation of ultrastructural details nicely shown in a review [46]. In addition, many authors described glycoproteins on nuclear membranes as well as on the viral envelope in the PNS, e.g. [4,11,47]. In addition, many tegument proteins were shown to be present within the nucleus and in perinuclear virions [48,49].

HSV-1∆Us3 virions accumulate in the PNS leading to the assumption that Us3 plays a significant role in de-envelopment [16,18]. In fact, 97.7% of the total enveloped R7041(∆Us3) virions were located in the PNS by 24 hpi indicating that their transport out of the PNS was severely disturbed. Interestingly, progeny R7041(∆Us3) virus are infective which has been repeatedly shown for Us3deletionmutants [16,18,20,21,50,51]. Therefore, the conclusion must be drawn that (i) R7041(∆U_S_3) virions in the PNS are infective; (ii) 98% of the total virus yield are enveloped by nuclear membranes; (iii) they contain all essential proteins; and (iiv) the processes of de- and re-envelopment are not essential for the R7041(∆U_S_3) mutant to become infective.

### 4.2. Virus Translocation to the Cytoplasm

To understand the translocation of virus particles from the PNS into the cytoplasm it has to be borne in mind that wild type HSV-1 virions can intraluminally transported into RER cisternae adjacent to the PNS [1,4,10,12,14,38,52,53,54,55] travelling long distances [29]. Intraluminal transportation was obviously inhibited in R7041(∆U_S_3) cells. Yet, if virions have the ability of intraluminal transportation it will be difficult to understand how the viral envelope can fuse with the membrane the virion is transported along since mechanisms of fusion are diametrically opposed to those of intraluminal transportation. Golgi cisternae may contain one or many virions. These virions may have gained access to the cisternal lumen by budding at its membrane or via intraluminal transportation form the RER provided RER membranes connect to Golgi membranes as shown in HSV-1 infected Vero cells [14]. Figure 7A also shows that the membrane of the outermost Golgi cisterna is a RER membrane, indeed. Direct connectivity between the PNS and Golgi cisternae has been shown in bovine herpes virus 1 infected MDBK cells [13]. Between the RER and the Golgi complex is the endoplasmic reticulum intermediate compartment (ERGIC) that connects to the RER [56]. Whether the ERGIC connects also to the Golgi complex remains to be elucidated, e.g., by cryo-electron microscopy [57] or by focused ion beam electron microscopy [58] followed by three dimensional reconstruction. A recent study employing high resolution confocal imaging suggest that the cis-Golgi approaches the RER and contacts the ER exit sites in the yeast Saccharomyces cerevisiae to capture cargo for transportation to the Golgi complex [59]. This ‘hug-and-kiss’ behavior could be another hypothetical route to transfer virions, which are intraluminally transported to ER exit sites, to the Golgi complex.

An alternative route for nucleus to cytoplasm capsids translocation has been shown to be via impaired nuclear envelope [6,60] and impaired nuclear pores [14,33,34]. Hence, there is good reason to assume that R7041(∆Us3) capsids may have gained access to the cytoplasm rather via impaired nuclear envelope than by de-envelopment. Believing in the de-envelopment theory, one could also argue that the fusion ability of the viral envelope with the ONM [18] was not entirely impaired.

### 4.3. Conclusion

In the absence of Us3, budding of capsids at the INM leads to infectious virions, which are almost entirely retained in the PNS, indicating that de- and re-envelopment are not essential to acquire the essential proteins for production of infective progeny virus. The data also indicate that Us3 is involved in intraluminal transportation of virions out of the PNS into the RER leading to the hypothesis that intraluminal transportation might be a major pathway in herpes virus egress.
